# Metagenome of a Microbial Community Inhabiting a Metal-Rich Tropical Stream Sediment

**DOI:** 10.1371/journal.pone.0119465

**Published:** 2015-03-05

**Authors:** Patrícia S. Costa, Mariana P. Reis, Marcelo P. Ávila, Laura R. Leite, Flávio M. G. de Araújo, Anna C. M. Salim, Guilherme Oliveira, Francisco Barbosa, Edmar Chartone-Souza, Andréa M. A. Nascimento

**Affiliations:** 1 Departamento de Biologia Geral, Instituto de Ciências Biológicas, Universidade Federal de Minas Gerais, Belo Horizonte, Minas Gerais, Brazil; 2 Centro de Pesquisas René Rachou Fundação Oswaldo Cruz, Belo Horizonte, Minas Gerais, Brazil; University of Oklahoma, UNITED STATES

## Abstract

Here, we describe the metagenome and functional composition of a microbial community in a historically metal-contaminated tropical freshwater stream sediment. The sediment was collected from the Mina Stream located in the Iron Quadrangle (Brazil), one of the world’s largest mining regions. Environmental DNA was extracted and was sequenced using SOLiD technology, and a total of 7.9 Gbp was produced. A taxonomic profile that was obtained by comparison to the Greengenes database revealed a complex microbial community with a dominance of *Proteobacteria *and *Parvarcheota. * Contigs were recruited by bacterial and archaeal genomes, especially *Candidatus Nitrospira defluvii *and *Nitrosopumilus maritimus*, and their presence implicated them in the process of N cycling in the Mina Stream sediment (MSS). Functional reconstruction revealed a large, diverse set of genes for ammonium assimilation and ammonification. These processes have been implicated in the maintenance of the N cycle and the health of the sediment. SEED subsystems functional annotation unveiled a high degree of diversity of metal resistance genes, suggesting that the prokaryotic community is adapted to metal contamination. Furthermore, a high metabolic diversity was detected in the MSS, suggesting that the historical arsenic contamination is no longer affecting the prokaryotic community. These results expand the current knowledge of the microbial taxonomic and functional composition of tropical metal-contaminated freshwater sediments.

## Introduction

Prokaryotic species exhibit broad distribution, having been researched across a wide range of natural environments such as soil, marine and freshwater, as well as in plants, animals and humans. Many of these species have been revealed to be important for the health and/or ecological balance of various environments. Indeed, a link between the set of microbial species and the host or environment-associated biological processes and health has been extensively reported [1, 2]. Because of their essential roles in life and in ecosystem functioning, ambitious multidisciplinary efforts across the globe are ongoing to characterize microbial communities [3].

Sediment has been recognized as a special realm in aquatic ecosystems because its species richness is higher than that of the water community and is comparable to soil microbial diversity [4, 5]. In mining-contaminated regions, sediments of water bodies play an important role in the transport and storing of contaminants. Indeed, sediment characteristics determine the ecological balance and biodiversity of the aquatic ecosystem [6].

There is a consensus in the literature that metal-contaminated freshwater sediment exhibits an extremely complex and well-adapted community [7–9]. These studies revealed that *Proteobacteria*, especially *Beta-proteobacteria*, and *Bacteroidetes* are the main contributors to the composition of these environments. It should be noted that sediment communities play an important role in biogeochemical cycling and are involved in the transformation of nutrients such as N and C [9].

Although previous studies of microbial communities in metal-contaminated freshwater sediment have been performed [5, 8, 10, 11], none of them assessed the microbial community of a metal-contaminated tropical sediment through taxonomic and functional diversity evaluation. Moreover, all of the studies, except Reis *et al*. [8], focused their analysis on sediments of temperate streams. However, due to the restricted power of the methodology employed by Reis *et al*. [8], these authors did not cover all of the taxonomic richness present in the tropical stream studied here. Thus, much is still unknown about the functional and taxonomic microbial diversity of tropical metal-contaminated streams. Considering that microorganisms play an essential role in environmental biogeochemical cycling, and may influence the speciation and bioavailability of metals, it is relevant to obtain a more comprehensive knowledge of the taxonomic and functional diversity of the prokaryotic community in metal-contaminated freshwater sediments.

One powerful strategy to assess both the functional and taxonomic microbial diversity is a metagenomic approach. Indeed, over the last 20 years, new sequencing technologies, together with metagenomic and computational tools, have transformed microbial ecology research. Metagenomics provides insight into the interactions of microbial communities with the environment and offers an extraordinary opportunity to comprehensively examine the ecosystem’s response to environmental changes [12]. However, metagenomic surveys that thoroughly assess the microbial diversity in freshwater sediments with extreme geochemical conditions involving high concentrations of As, Fe, and Mn are still lacking.

In this study, we applied a shotgun metagenomic approach and a metabolic analysis to examine the taxonomic and functional composition of the prokaryotic community of a historically metal-contaminated tropical stream sediment. The stream studied herein, the Mina Stream, is located in the Iron Quadrangle (IQ, Brazil), one of the world’s largest mining regions, which has been undergone to mining activities since the late 17th century. Accordingly, the IQ presents a historical metal contamination of waters and sediments from streams and rivers, including the Mina Stream [8, 13–16]. We also performed comparative metagenomic analysis between our metagenome and a rich arsenic well metagenomic dataset from Bangladesh [17].

## Material and Methods

### Ethics statement

For sampling in the Mina Stream, no specific permit was required for the described field study. The study location is not privately owned or protected in any way, and we confirmed that the field study did not involve endangered or protected species.

### Study area

The Mina stream (19°58’46.80”S and 43°49’17.07”W) is located in one of the world’s largest mining regions and is extremely rich in iron and gold ores (Iron Quadrangle, Minas Gerais state, Brazil). Collections of Mina stream sediment have been previously described by our group [11]. This stream was chosen because it has suffered stress by metal pollution exceeding the maximum allowable concentrations established by Brazilian environmental regulations, such as Cu 387.7 mg kg^1^, Zn 180.9 mg kg^1^ and As 297.1 mg kg^1^, which were presented in an earlier study [11].

The sediment sample in this study was taken from the upper part (oxic zone) during the dry season and was named according to the location from which it was retrieved, i.e., Mina Stream sediment (MSS). For metabolic analysis, the anaerobic environment of the sediment sample was maintained by substituting the O_2_ for CO_2_ using a CO_2_ pump, and the tube was hermetically closed. Two hours after collection, the sediment sample was introduced into an anaerobic chamber where subsequent experiments were performed.

### Microbial metabolic diversity

The capability of aerobic and anaerobic sediment microbial communities to utilize different carbon sources was assessed using Biolog Ecoplate (Biolog.Inc, Hayward, CA, USA). This system contained 31 carbon sources, in triplicate, divided into amines, amino acids, carbohydrates, carboxylic acids, and polymers, among others. In addition to the specific carbon source, each well contained tetrazolium violet redox dye as a color indicator for the utilization of the carbon sources by the microorganisms [18] (S2 Table). Sediment sample was filtered (10 g wet weight; pore size 0.45μm) and diluted in sterile saline. Then, 120 μL from the 10^-2^ dilutions was inoculated into each well and subsequently incubated aerobically and anaerobically in the dark at 28°C. Color development was measured at OD_590_ every 24 h for 4 d using an ELISA plate reader (BIO-RAD Model 3550 Microplate Reader). For the anaerobic BIOLOG assay, four plates were used, one for each day of reading. This procedure was performed by taking into account the loss of anaerobic conditions when the plate was withdrawn from the anaerobic chamber. For aerobic conditions, one plate was used. The detected value of the absorbance for the blank (water) reading was subtracted from all wells.

### Ecoplate data analysis

The data generated by 96 h readings were statistically analyzed. Because raw OD_590_ values were corrected, the microbial activity for each microplate was expressed as the average well-color development (AWCD) and was calculated as follows: *AWCD = Σ0DI/31* where ODi is the optical density value for each well. The richness (number of carbon substrates consumed) and the Shannon-Weaver index were calculated using a cutoff line of OD = 0.25 for a positive microbial response [19]. The Shannon-Weaver index was calculated as follows: *H*′ = -*Σpi (ln pi)*, where pi is the ratio between the microbial activity of each substrate (ODi) and the sum of microbial activities of all substrates (ΣODi). The Evenness index was calculated with the formula *E = H′/ln R*, where H′ is the value of the Shannon index, and R is the richness of substrates.

### DNA extraction and shotgun metagenomic sequencing

Total DNA was extracted from the sediment sample (10 g wet weight) using the PowerSoil DNA Extraction kit (MoBio Laboratories, USA) according to the manufacturer’s instructions. Quantification and quality of total DNA were determined using the Agilent 2100 Bioanalyzer equipment according to the manufacturer’s instructions.

Sediment sample was subjected to shotgun sequencing using the high-throughput sequencer Applied Biosystems SOLiD v.4 following the manufacturer’s protocol. Briefly, 10 μg of total DNA was randomly fragmented using the Covaris S2 System. A DNA fragment library from 200 to 250 bp long was constructed for sequencing. Then, emulsion PCR was performed to clonally amplify fragments on sequencing beads, followed by enrichment and preparation for deposition in plate for sequencing according to the manufacturer’s instructions (http://tools.lifetechnologies.com/content/sfs/manuals/SOLiD4_Library_Preparation_man.pdf). After sequencing, 50 bp reads were generated for further analysis.

### 16S rRNA gene amplification and sequencing

Amplification of the V3-V4 hypervariable region of the 16S rRNA gene was performed using region-specific bacterial/archaeal primers S-D-Bact-0341-b-S-17 forward 5’-CCTACGGGNGGCWGCAG-3’ and S-D-Bact-0785-a-A-21 reverse 5’-GACTACHVGGGTATCTAATCC-3’ [20], with Illumina adapters added. Barcoded amplicons were generated using KAPA HiFi HotStart ReadyMix (KAPA, Woburn, MA, USA) and were purified using AMPure XP beads (Agencourt Bioscience, Beverley, MA, USA). Sequencing was performed using the MiSeq platform (Illumina, Inc., San Diego, CA, USA) according to the manufacturer’s instructions (http://support.illumina.com/documents/documentation/chemistry_documentation/16s/16s-metagenomic-library-prep-guide-15044223-b.pdf).

### Bioinformatics analysis

#### V3-V4 region from 16S rRNA gene

16S rRNA microbiota primary data analysis was performed with PRINSEQ (stand alone lite version, http://prinseq.sourceforge.net/), where quality-based trimming was performed. Reads with N's or an overall mean Q-score of < 25 were discarded. The resulting fasta file was also screened for ambiguous bases and homopolymers using MOTHUR v.1.33.0 (http://www.mothur.org). Furthermore, chimeras were detected using the UCHIME algorithm (http://drive5.com/uchime).

Operational taxonomic units (OTUs) and taxonomic classification were determined using the MOTHUR pipeline [20, 21] and the Greengenes reference database (http://greengenes.secondgenome.com/downloads/database/13_5, from May 2013) to obtain the microbial composition of the MSS microbiota. OTUs were determined using similarity levels between sequences of at least 97% for classifying a microorganism at the species level, as proposed by Drancourt *et al*. [22]. Good’s coverage [23] was calculated for OTUs with an evolutionary distance of 0.03. Rarefaction curves were calculated for OTUs with an evolutionary distance of 0.03, 0.05 and 0.10. The nucleotide sequences were submitted to Sequence Read Archive (SRA, http://www.ncbi.nlm.nih.gov/sra/) with the accession number of SRR1573431.

#### Shotgun metagenome data

Metagenomic primary data analysis was performed with SOLiD Accuracy Enhancement Tool (SAET) software (http://solidsoftwaretools.com/gf), a spectral alignment algorithm that screens for errors inherent to the sequencing platform and the encodeFasta.py program (http://gnome.googlecode.com/svn/trunk/pyGenotypeLearning/src/pytools/encodeFasta.py), that converts the sequences represented in color space to letter space format. Then, the assembly of the metagenome data was performed to generate contigs using the Metavelvet software [24] with parameters according to the recommendations of the authors (kmer 27,-exp_covauto) [24].

A Fasta file with contig sequences was deposited into the Metagenomics RAST Server (MG-RAST v3.3) [25]. Prior to annotation, MG-RAST provides a quality control of sequences that consists of artificially removing duplicate sequences and screening based on quality and size of sequences. Functional analysis was performed using the SEED subsystem and KEEG available on MG-RAST with the following cutoff parameters: 1x10^-5^ e-value and 60% of identity percentage [26]. The data from this study are available via MG-RAST with the ID 4519449.3.

A recruitment plot was used to identify abundant species genomes in the MSS metagenome. In this representation, MSS metagenome contigs were compared to individual bacterial genomes. Fragment recruitment of the MSS contigs was performed using BLASTN against bacterial and archaeal complete genomes. Data were plotted using R (http://cran.r-project.org), and the criteria for counting a hit were a minimum identity of 90%, e-value cutoff 0.001 and minimum alignment of 50 bp.

#### Comparative metagenomic analysis

Comparative metagenomic analysis was performed using the Statistical Analysis of Metagenomic Profiles (STAMP) program [27] to determine statistically significant functional composition differences in any two metagenomes using two-sided Fisher exact tests [27]. The most important metabolic categories were selected by using a p-value >0.05. To accomplish that, the MG-RAST functional matches at all levels were compared using the SEED database (http://www.theseed.org). The statistical comparison was conducted with the data from a rich arsenic well metagenome (4461675.3) [17] due to similarity between the two environments, i.e., the high As contamination.

### Real-time PCR (qPCR)

Quantitative real-time PCR was performed to estimate the absolute number of copies of bacterial and archaeal 16S rRNA genes in the MSS. To accomplish this outcome, total DNA sample was added to a 20 μl reaction containing a SYBR Green master mix and the bacterial and archeal primer set: 338F (5’-TACGGGAGGCAGCAG-3’) and 344F (5'-ACGGGGCGCAGCAGGCGCGA-3’), respectively [28] and 518R (5’-ATTACCGCGGCTGCTGG-3’) for both [29]. Standard curves were generated from the 16S rRNA gene amplicons obtained using conventional PCR from *Halococcus morrhuaea* ATCC 17082 and *Escherichia coli* ATCC 25922 as previously described by Cardinali-Rezende *et al*. [30]. The procedure was performed using the ABIPRISM 7900HT sequence detection system (Applied Biosystems, Foster City, CA). The conditions used to amplify the 16S rRNA gene from bacteria and archaea were according to Cardinali-Rezende *et al*. [30].

## Results

### Taxonomic composition of the prokaryotic community

The MSS microbiota resulted in 273,710 high-quality reads with an average read length of 450 bp. Of a total of 31,656 OTUs, 678 OTUs were not classified within the Bacteria and Archaea domains. Thus, a total of 30,978 OTUs remained for downstream analysis. Of these OTUs, 22,184 were singletons and 2,242 were doubletons composed of only a few reads (27,077). Bacteria were by far the most abundant prokaryotic domain, constituting 98.2% (30,738 OTUs), whereas archaeal reads showed a relative paucity (1.8%, 240 OTUs). The Good’s coverage value (89%) and rarefaction curve (S1 Fig.) obtained with an evolutionary distance of 0.03 indicated that most of the prokaryotic diversity was detected in the sample.

**Fig 1:**
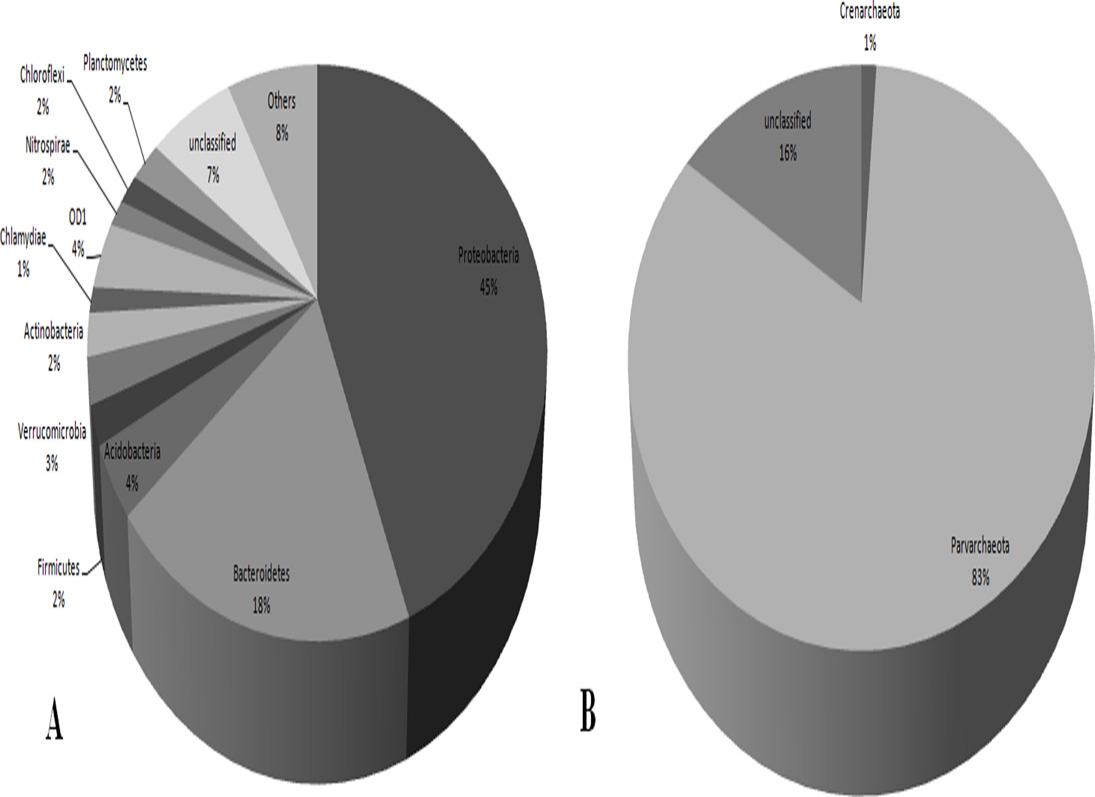
Bacterial and archaeal phyla abundance. Taxonomic composition of bacterial (A) and archaeal (B) taxa from MSS microbiota based on the Greengenes database. Other bacteria: *Gemmatimonadetes*, *Cyanobacteria*, *OP3*, *OP11*, *Spirochaetes*, *TM7*, *Chlorobi*, *WS3*, *Elusimicrobia*, *GN04*, *TM6*, *GN02*, *Tenericutes*, *Armatimonadetes*, *BRC1*, *NC10*, *WPS*-2, *Fibrobacteres*, *Fusobacteria*, *H*-*178*, *FCPU426*, *Kazan*-3B-28, *WS5*, *NKB19*, *Thermi*, *AC1*, *TPD*-58, *WS6*, *Synergistetes*, *OP8*, *WS2*, *ZB3*, *SC4*, *OP1*, *SBR1093*, *SR1*, *Lentisphaerae*, *GAL15*, *PAUC34f*, *LCP*-89 and *MVS*-104.

Bacterial and archaeal phyla diversity are shown in Fig. 1 and S1 Table. A total of 30,738 OTUs were assigned to 52 known bacterial phyla. Nevertheless, most OTUs were affiliated with four phyla: *Proteobacteria* (45%), *Bacteroidetes* (18%), and an equal proportion (4%) of *Acidobacteria* and OD1. The group “other bacteria” comprised minor bacterial phyla such as *Gemmatimonadetes*, *Cyanobacteria*, *OP3*, *OP11*, *Spirochaetes*, and *TM7*, among others, representing 8% of the OTUs. Furthermore, 2,157 OTUs were considered to be unclassified at the phylum level and, thus, may represent new bacterial taxa (Fig. 1).

All proteobacterial classes were represented, with the *Beta*-, *Gamma*-, and *Deltaproteobacteria* classes being the most abundant (81%). *Bacteroidetes* were identified primarily as members of the *Sphingobacteria* (53%), *Flavobacteria* (17%), *Bacteroidia* (17%) and incertae sedis (13%) classes. The *Acidobacteria* phylum was represented by 19 classes, with Gp6, Gp17, Gp3 and *Holophagae* accounting for 69% of representation.

Only 8,430 OTUs (26.6%) were classified at the genus level. The predominant genera observed were, *Sediminibacterium* (*Bacteroidetes*, 520 OTUs), *Flavobacterium* (*Bacteroidetes*, 392), *Prevotella* (*Bacteroidetes*, 371), *Geobacter* (*Proteobacteria*, 323), *Nitrospira* (*Nitrospirae*, 303), *Haliscomenobacter* (*Bacteroidetes*, 250), *Thermomonas* (*Proteobacteria*, 245), *Thiobacillus* (*Proteobacteria*, 240), *Acinetobacter* (*Proteobacteria*, 222) and *Acidovorax* (*Proteobacteria*, 200).

**Fig 2:**
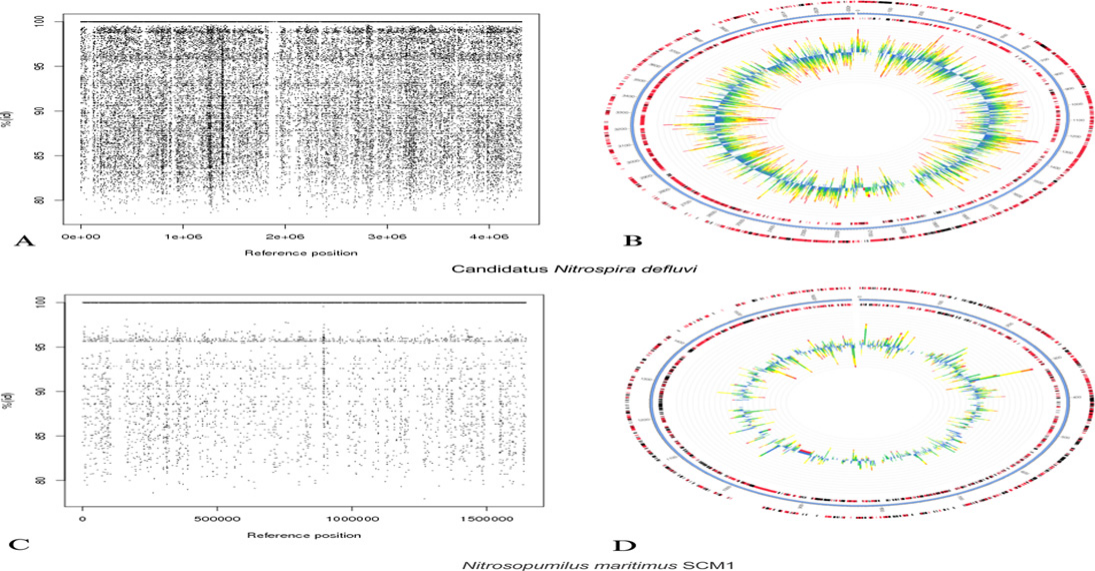
Fragment recruitment plots of the MSS contigs. Candidatus *Nitrospira defluvi* (A and B—FP929003.1) and *Nitrosopumilus maritimus* (C and D—CP000866.1). The comparison was made using BLASTn. Vertical axis showed the % identity of the metagenomic contigs to the respective bacterial or archaeal genome. A and C—recruitment by R software; B and D—recruitment by MG-RAST.

Recruitment of MSS metagenome contigs by bacterial genomes are illustrated in Fig. 2 and S2A-G Figs. The bacterial genome that recruited the majority of the contigs was Candidatus *Nitrospira defluvii* (Fig. 2A and B). Other bacterial species were also reasonably well recruited, such as *Anaeromyxobacter dehalogenans*, *Chitinophaga pinensis* DSM2588, *Geobacter metallireducens*, *Leptothrix chlolodnii*, *Sideroxydans lithotrophicus*, *Thiobacillus denitrificans*, and *Thiomonas* 3As (S2 Fig.).

The taxonomic affiliation of the Archaea domain revealed that most of the OTUs belonged to the *Parvarchaeota* phylum (83%) represented by the *Parvarchaea* (83%) and *Micrarchaea* (17%) classes. The *Crenarchaeota* phylum (1%) was also represented by three OTUs related to the Miscellaneous Crenarchaeotal Group (MCG). Although members of the *Thaumarchaeota* phylum were not identified in the MSS microbiota, it was possible to recruit the partial genome of three *Thaumarchaeota* species: *Nitrosopumilus maritimus* SCM1, an ammonia oxidizing archaea belonging to the *Nitrosopumilaceae* family that was originally isolated from a marine fish tank [31] (Fig. 2C and D); *Cenarchaeum symbiosum*, a psychrophilic archaea species that belongs to *Cenarchaeaceae* family and inhabits a marine sponge; and Candidatus *Nitrososphaera gargensis*, an ammonia oxidizing species from *Nitrososphaeraceae* family (S2H-I Figs.).

### Abundance of the bacteria and Archaea domains

The absolute quantification of bacterial and archaeal communities by qPCR was accomplished and generated R^2^ values of 0.99 for both curves and slopes of -3.23 and -3.35, respectively (S3A-D Figs.). According to qPCR analysis, the bacterial 16S rRNA gene copy number (7.7 x 10^6^ gene copies g^−1^) was two orders of magnitude higher than the archaeal, with 5.3 x 10^4^ gene copies g^−1^ in the sediment sample (S4A and B Figs.).

### Overview of metagenomic data

Random shotgun metagenome sequencing from MSS resulted in 158,882,631 reads (50 bp per read) totaling a ~7.9 Gbp dataset. Assembly of reads by Metavelvet resulted in 378,588 contigs ranging from 60 to 2911 bp. After being trimmed by MG-RAST based on quality, size, and artificial removal of duplicate reads, a total of 350,111 clean contigs were used for further analysis. The contig dataset was used to determine the functional analysis. The MSS metagenome exhibited a wide range of GC content from 15% to 80%. Most of the contigs were grouped and ranged from 40 to 60% GC content, with an average GC content of 45 ± 8%.

### SEED and KEEG analyses with MG-RAST

Of the 350,111 contigs analyzed for the functional annotation based on the SEED subsystem classification (MG-RAST), 135,632 contigs (39%) could be assigned to functional categories, i.e., predicted proteins with known functions. Nevertheless, most of the contigs (53%) were related to predicted proteins with unknown function, whereas the remaining contigs (8%) presented no match with the SEED database.

**Fig 3:**
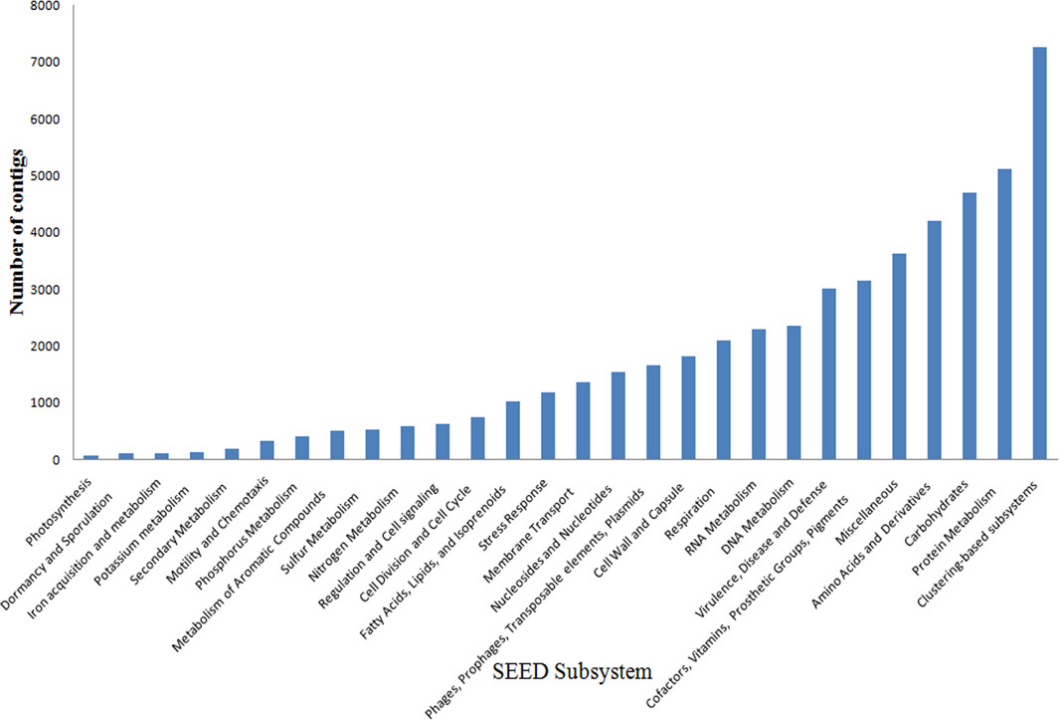
SEED subsystems distribution of the MSS metagenome based on MG-RAST annotation. The cutoff parameters were e-value 1x10^-5^ and 60% of identity.

Twenty-eight functional subsystems were identified in the MSS metagenome. Protein metabolism, clustering-based subsystems, miscellaneous, carbohydrates, and RNA metabolism presented the largest number of annotated contigs. Other subsystems were related to mobile elements (phages, transposons, integrons, plasmids, and pathogenicity islands) (4%) and stress response (3%), both of which are involved in the fast response and adaptation of the microbial community to changes in the environment (Fig. 3).

Functional analysis with the KEGG Mapper tool of the MG-RAST allows an integrated view of the environmental global metabolism. Assignment of the MSS contigs revealed that most of the metabolic pathways were detected (data not shown). The metabolic pathways identified in the KEGG database as the most abundant were carbohydrate, amino acids, and energy metabolic pathways, indicating that microbial communities inhabiting the MSS are well adapted to degrade carbon substrates such as soluble carbohydrates or polysaccharides and amino acid and derivatives.

Among the genes detected in the MSS, we focused our SEED and KEGG analyses on metal resistance and nitrogen metabolism, which might have particular importance for this environment.

### Nitrogen metabolism analysis

The Mina Stream is a eutrophic water body presenting high nitrogen concentration and of its inorganic forms [11]. Therefore, nitrogen metabolism was analyzed, and revealed the presence of enzymes that play a role in ammonia assimilation (49%), nitrate and nitrite ammonification (33%), allantoin utilization (7%), nitrogen fixation (5%), nitric oxide synthase (3%), and cyanate hydrolysis (3%). Relevant genes involved in these six processes revealed by SEED and KEGG databases are displayed in Table 1 and S5 Figs.

**Table 1:** The most frequent nitrogen metabolism and metal resistance proteins in the MSS metagenome obtained using the MG-RAST web server based on SEED database.

	Protein	Number of contigs
**Nitrogen Metabolism**		
Allantoin Utilization	2-hydroxy-3-oxopropionate reductase (EC 1.1.1.60)	43
	Allantoate amidohydrolase (EC 3.5.3.9)	50
	Ureidoglycolate dehydrogenase (EC 1.1.1.154)	53
Ammonia assimilation	Ammonium transporter	64
	Ferredoxin-dependent glutamate synthase (EC 1.4.7.1)	525
	Glutamate synthase [NADH] (EC 1.4.1.14)	43
	Glutamate synthase [NADPH] large and small chain (EC 1.4.1.13)	123
	Glutamate-ammonia-ligase adenylyltransferase—GlnE (EC 2.7.7.42)	44
	Glutamine synthetase type I and type III (EC 6.3.1.2)	130
Cyanate hydrolysis	Carbonic anhydrase—CynT (EC 4.2.1.1)	44
Nitrate and nitrite ammonification	Assimilatory nitrate reductase large subunit (EC:1.7.99.4)	77
	Nitrite reductase [NAD(P)H] small subunit (EC 1.7.1.4)	30
	Nitrite reductase probable electron transfer 4Fe-S subunit (EC 1.7.1.4)	210
	NrfC protein	51
	Polyferredoxin NapH (periplasmic nitrate reductase)	59
	Putative thiol:disulfide oxidoreductase, nitrite reductase complex assembly	76
	Respiratory nitrate reductase delta chain (EC 1.7.99.4)	42
	Respiratory nitrate reductase subunit, conjectural (EC 1.7.99.4)	78
Nitric oxide synthase	Manganese superoxide dismutase (EC 1.15.1.1)	42
Nitrogen fixation	AnfO protein, required for Mo- and V-independent nitrogenase	51
	Nitrogenase (vanadium-iron) beta chain (EC 1.18.6.1)	47
**Metal resistance**		
Arsenic resistance	Arsenic resistance operon (ArsB, ArsH, ArsA, ArsR, ArsD)	67
	Arsenical-resistance protein ACR3	112
	Respiratory arsenate reductase, Mo binding subunit and FeS subunit (ArrA and ArrB)	124
Cobalt-zinc-cadmium resistance	Cadmium-transporting ATPase—CRA (EC 3.6.3.3)	24
	Probable cadmium-transporting ATPase—PCT (EC 3.6.3.3)	20
	Cation efflux system protein (CusA,CusR, CusC, CusB)	990
	Cobalt-zinc-cadmium resistance protein (CzcA, CzcD, CzcB, CzrR, CzrB)	2007
	Probable Co/Zn/Cd efflux system membrane fusion protein (CusB/CzsB)	160
	Putative silver efflux pump	45
Copper homeostasis	Copper-translocating P-type ATPase (EC 3.6.3.4)	504
	Cytochrome c heme lyase subunit CcmF	31
	Multicopper oxidase	187
Mercuric reductase	FAD-dependent NAD(P)-disulphide oxidoreductase	85
Mercury resistance operon	Mercuric resistance proteins (MerC, MerE, MerT, MerD, MerR, MerP, MerA)	200
Resistance to chromium compounds	Chromate resistance proteins (ChrI, ChrA, ChrC)	17
Zinc resistance	Response regulator of zinc sigma-54-dependent two-component system (ZraR)	72

### Metal resistance analysis

The genes associated with heavy metals were highly diverse, with cobalt-zinc-cadmium (47%) and copper resistance (30%) being the most abundant, followed by the arsenic resistance genes accounting for 6% (Table 1). Interestingly, the presence of the *arsC* resistance gene was not detected in the MSS metagenome even though this gene is the most widespread arsenic resistance gene in the environment [32].

### Statistical comparison of As-contaminated environment

**Fig 4:**
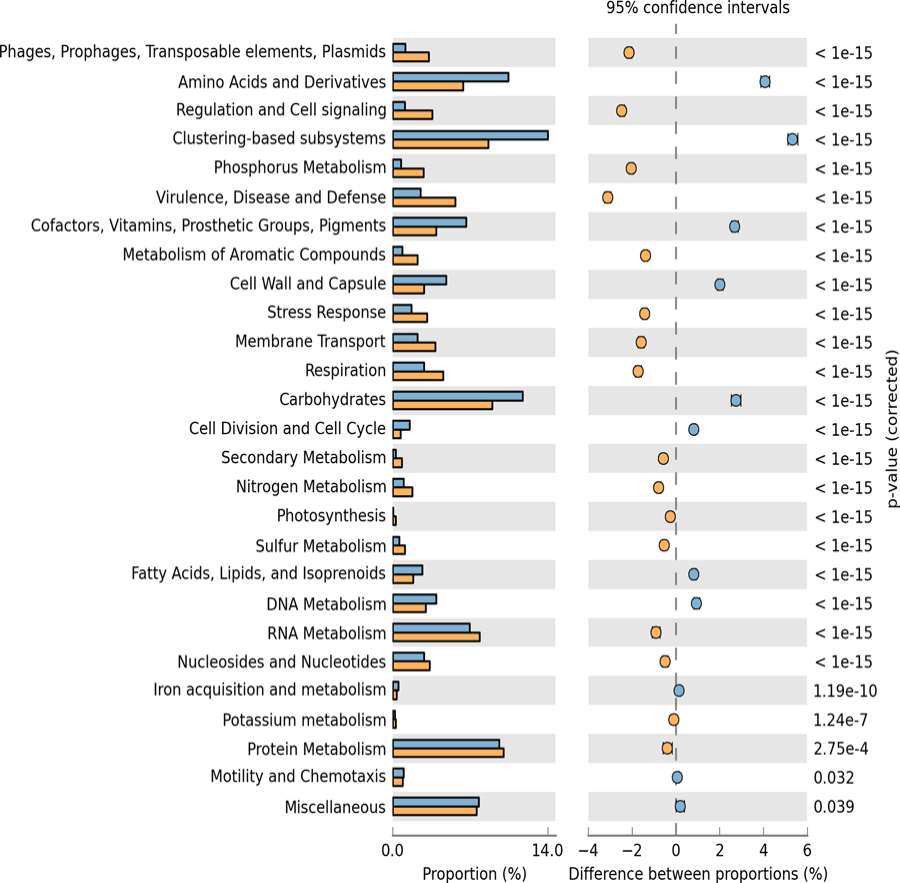
Significant SEED subsystem differences as a result of a Fisher exact test between the MSS and RAW metagenomes conducted with the STAMP program. Enrichment of SEED subsystem in the RAW metagenome has a positive difference between proportions (blue circles), whereas enrichment of SEED subsystem in the MSS metagenome has a negative difference between proportions (orange circles). Bars on the left represent the proportion of each subsystem in the data. Subsystems difference with a *p value* of >0.05 were considered to be significant.

**Fig 5:**
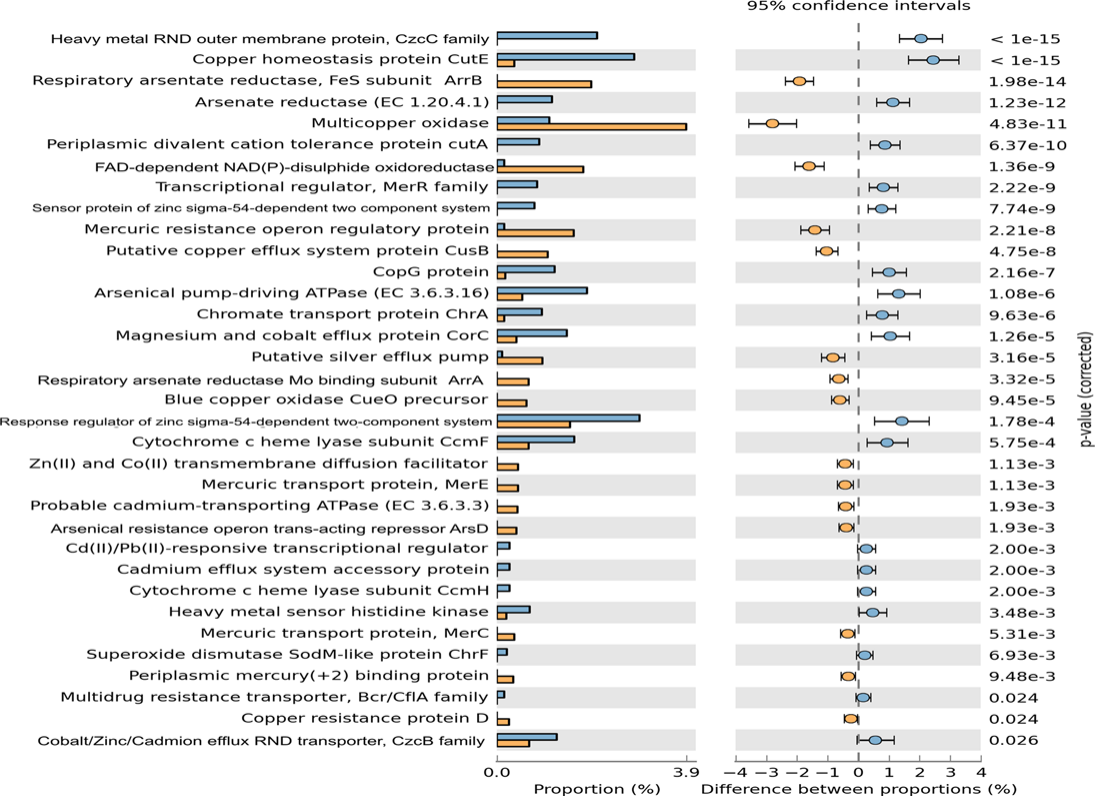
Significant metal resistance genes differences as a result of a Fisher exact test between the MSS and RAW metagenomes conducted with the STAMP program. Enrichment of metal resistance genes in the RAW metagenome has a positive difference between proportions (blue circles), whereas enrichment of metal resistance genes in the MSS metagenome has a negative difference between proportions (orange circles). Barson the left represent the proportion of each metal resistance protein in the data. Metal resistance difference with a *p value* of >0.05 were considered to be significant.

Statistical comparison of the SEED subsystem resemblances between two or more environments can reveal enriched subsystems for a particular environment. To determine biologically significant differences, the functional subsystems detected in the MSS metagenome were statistically compared with the RAW metagenome, as described by Mailloux *et al*. [17]. SEED subsystem comparison revealed a high degree of similarity between the MSS and RAW metagenomes (Fig. 4). However, some differences were observed with significantly over abundant reads in the MSS, which were assigned to mobile elements, regulation and cell signaling, phosphorus metabolism, virulence and defense subsystems, among others. By contrast, the RAW metagenome identified more reads in the amino acid and derivative, clustering-based, carbohydrates, and subsystems related to cell maintenance (Fig. 4). The two metagenomes, MSS and RAW, statistically differed in the enrichment of contigs related to respiratory arsenate reductase (ArrA and ArrB proteins) and multicopper oxidase, which were more frequent in the MSS. By contrast, the RAW metagenome overrepresented arsenate reductase (ArsC) and copper homeostasis (CutE) proteins in the dataset (Fig. 5).

### Metabolic diversity and community-level physiological profiles (CLPP)

The metabolic profile of the microbial community of the MSS was assessed using Biolog Ecoplate (Biolog, Inc.). Substrate utilization patterns from microbial communities are shown in S2 Table. The highest metabolic diversity was observed under anaerobic conditions (30 carbon sources consumed), whereas the community under aerobic conditions consumed 26 carbon sources. 2-hydroxy benzoic acid was the only carbon source not consumed by either microbial community. The substrates α-ketobutyric acid, L-threonine, glycogen, and α-D-lactose were not consumed by the aerobic microbial community.

**Fig 6:**
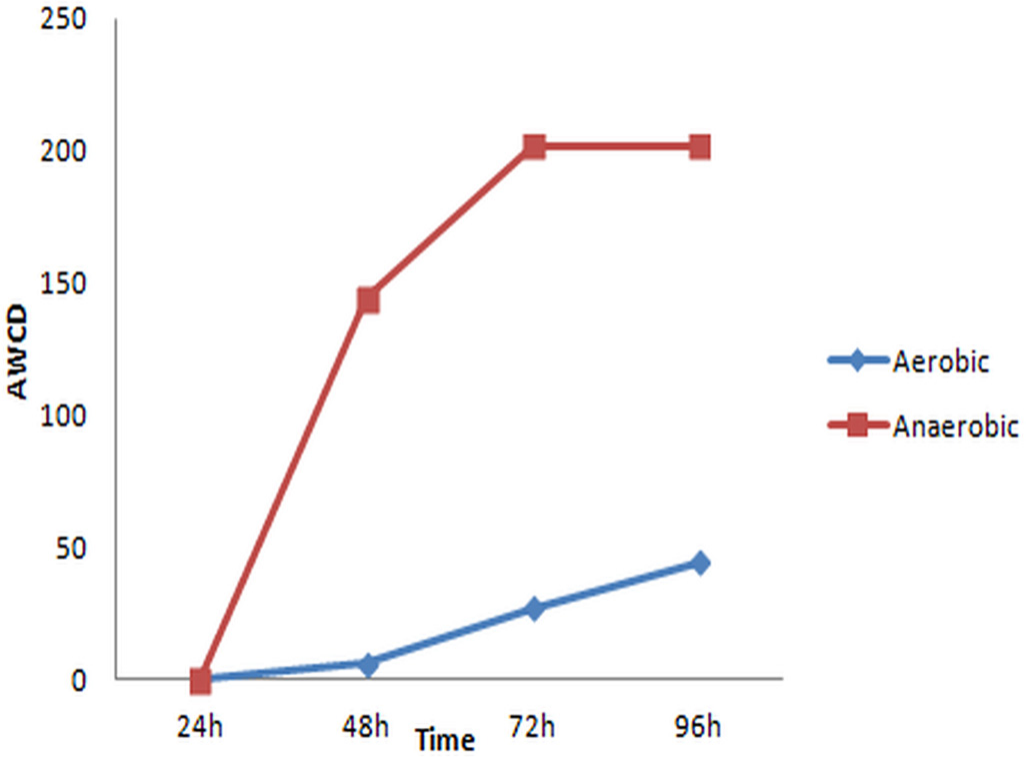
Average well-color development (AWCD) calculated from the consumption of carbon sources of anaerobic and aerobic microbial communities.

AWCD reflects the carbon source utilization ability of the microbial community over time. AWCD analysis showed that the microbial community under anaerobic conditions reached the maximum carbon source utilization at just 72 h, after which time the activity reached a plateau (as demonstrated by the maximum color development). By contrast, the microbial community under aerobic conditions did not reach the maximum color development, showing slower growth and consumption of the carbon source (Fig. 6).

The Shannon and Simpson diversity indices of the microbial community metabolic profile were calculated, revealing moderate diversity in both communities (S2 Table). Although the anaerobic community presented greater diversity, the differences were not statistically significant (*P* ≤ 0.05) (S2 Table). In addition, the Simpson's index of microbial community response showed that a few dominant microbial species were responsible for the metabolic profile of both communities.

## Discussion

The microbial community plays an important role in the freshwater environment, especially in stream ecosystems where they are responsible for most of the organic matter decomposition [33]. The dataset presented in this study is the first to taxonomically and functionally characterize the microbial community of a metal-contaminated sediment from a tropical freshwater stream using a combination of approaches such as metabolic fingerprinting, qPCR, and shotgun metagenomic sequencing.

Taxonomic analyses revealed that a highly complex bacterial community was present in the MSS. Taxonomic data indicated *Proteobacteria* (especially *Beta-proteobacteria*) was the most abundant phylum followed by *Bacteroidetes*. A previous investigation on the prokaryotic diversity in the MSS also showed the predominance of the *Proteobacteria*, but with its classes presenting different tendencies, and *Bacteroidetes* phyla [8]. However, the present study revealed that the bacterial and archaeal 16S rRNA gene copy number was lower in the dry season, in contrast to the increase detected in the rainy season by Reis *et al*. [8]. The observed increase, up to 10 times that of metal concentrations (mainly Zn and As), in the dry season may have affected the cell abundance of the microbial communities present in the MSS, a finding that was reflected in the abundance of the 16S rRNA gene copy number. The eutrophic environment and the presence of high concentrations of metals in the MSS could explain the predominance of *Beta-proteobacteria* and *Bacteroidetes*. Indeed, according to Brümmer *et al*. [33], the predominance of *Beta-proteobacteria* is associated with the presence of high concentrations of ammonia and metals in contaminated water.

Our freshwater tropical sediment results differ from those reported recently for temperate sediments showing that *Proteobacteria* (especially *Deltaproteobacteria*) and *Acidobacteria* were the most abundant phylas [34]. In addition, *Bacteroidetes* were found to be in low proportion in freshwater sediment, albeit enriched when interdital wetland sediments were analyzed [34]. It should be noted that our data presented a taxonomic similarity with a previous investigation in tropical pristine sediment [8].

Several bacterial species that play an important role in metal contaminated environments were found to inhabit the MSS, as supported by the recruitment plots (Fig. 2 and S2A-E Figs.). The *Beta-proteobacteria* class harbors chemolithoautotrophic members as ferrous iron oxidizing bacteria (FeOB), which were broadly represented in our data [35]. The *Gallionellaceae* family was represented by *Sideroxydans lithotrophicus*, a neutrophilic FeOB that prefers low oxygen and iron-rich environments [7]. *S*. *lithotrophicus* may play an important role in the removal of As from the MSS environment as FeIII binds with arsenate (AsV), which facilitates its precipitation and decreases its bioavailability in the environment [7]. *Leptothrix chlolodnii*, which is often found in eutrophic freshwater environments, was detected in our analysis. This bacterium oxidizes MnII into manganese oxide (MnIII and MnIV) [36, 37]. The *Betaproteobacteria* found in our sample included, among others, *Thiobacillus denitrificans* and *Thiomonas cuprina*. The former oxidizes various reduced inorganic sulfur compounds, such as ferrous sulfide (FeS), coupling with the reduction of nitrate [38, 39]. The latter is an AsIII-oxidizing bacterium that is ubiquitous in arsenic-contaminated environments and is capable of gaining energy from the oxidation of reduced inorganic sulfur compounds (e.g., able to perform the dissimilatory oxidation of iron) [39, 40]. Three FeIII-reducing members of the *Deltaproteobacteria* class were detected. One of them, *Anaeromyxobacter dehalogenans*, is a dissimilatory FeIII-reducing bacterium known to gain energy with Fe reduction [41], a contrasting role to that performed by *Thiomonas cuprina*. The two other members belonged to the *Geobacter* genus and showed the highest abundance among the *Deltaproteobacteria* of the MSS metagenome. Members of this genus were the most recovered in enrichment cultures by FeIII reduction [42]. Altogether, the presence of these taxa may reflect the high concentrations of metals such as Fe, Mn, Cu, As, and Zn found in the MSS. Moreover, the genome of *Chitinophaga pinensis* was well represented in the fragment recruitment plots [43]. This species is associated with organic carbon cycling in both anaerobic and aerobic sediments through the breakdown of simple carbohydrates to organic acids and degradation of a wide range of biopolymers [44, 45].

Members of *Actinobacteria*, *Firmicutes*, and *Nitrospirae* are generally recovered in large proportions from freshwater environments [46, 34], which is in contrast to the present observation for the MSS. Studies suggest that the abundance of the *Actinobacteria* and *Firmicutes* phyla is significantly correlated with metal-contaminated environments, particularly resistance to As and Hg [7, 8, 47, 48]. However, the metal contamination found in MSS does not appear to favor their abundance. Future research will be needed to ascertain the reason for the observed decrease of the abundance of these bacteria in this freshwater sediment and to find whether it is a widespread phenomenon.

The members of the Archaea domain from the MSS belonged to the *Parvarchaeota* and *Crenarchaeota* phyla. The *Crenarchaeota* phylum has been previously described in metal-contaminated environments [7, 49–51]. Our data contrasted with previous studies on archaeal diversity in metal-impacted environments that usually find a predominance of *Crenarchaeota* [8, 52]. The *Parvarchaeota* phylum was recently proposed by Rinke *et al*. [53] from single-cell genome sequencing of an uncultured archaea. *Thaumarcheota* were represented, only in the metagenomic shotgun sequencing data, by the following ammonium oxidizer species: *Cenarchaeum symbiosum*, *Nitrosopumilus maritimus*, and Candidatus *Nitrosphaera gargensis* [54–56]. Previous investigation on water columns of the Amazon River also detected *Cenarchaeum symbiosum* and *Nitrosopumilus maritimus*, indicating the importance of these species in the nitrogen (N) cycle of sediment from freshwater environments [50, 57]. It should be noted that the species *Nitrosopumilus maritimus*, detected in the MSS metagenome, showed the highest genome coverage of archaeal reads, indicating that this chemolithoautotrophic nitrifier is globally distributed and is essential for the nitrification mechanisms in this environment.

The presence of various metal resistance genes detected in the MSS metagenome was expected, because the MSS exhibited high concentrations of As, Mn, Zn, and Cu. Despite absence of Co and Cd in MSS, resistance genes associated with cobalt-zinc-cadmium resistance were the most abundant. Resistance determinants to these metals are usually organized as an operon harboring the genes *czcC*, *czcB*, and *czcA*, which are responsible for expression of an efflux pump that transports the ions Co^+2^, Zn^+2^, and Cd^+2^ out of the bacterial cell [58, 59]. A previous study investigated the expression of this operon in the presence of these metals separately and found that the expression was more efficient in the presence of high concentrations of Zn [60]. Thus, the high concentration of Zn in the MSS could explain the abundance of these genes in this environment. Moreover, genes that confer resistance to Hg were also found in MSS, despite the low concentration of this metal (<2.5 mg kg^-1^ and <0.1 mg l^-1^ for sediment and water, respectively). This finding could be due to the fact that the Hg resistance genes are co-selected as they are usually located on plasmids and transposons that harbor other resistance genes, such as resistance to betalactamic antibiotics, kanamycin, tetracycline, and others [61–63].

The Cu resistance gene, the second most abundant in the MSS, may be related to bacterial cell protection mechanisms against high concentrations of this metal found in this environment. Cu is an essential metal for the metabolism of the cell, because it is required as a cofactor for several enzymes [64]. Nevertheless, high concentrations of this metal may be toxic for the bacterial cells that have developed homeostasis mechanisms to ensure appropriate internal concentrations of Cu [65].

The As resistance mechanism most widespread in the environment, performed by the ArsC enzyme, was not detected in the MSS. Interestingly, the other genes of the *ars* operon (*arsA*, *arsB*, *arsD*, *arsH*, *arsR*) were found. The bacterial respiratory arsenate reductase enzymes encoded by the *arrA* and *arrB* genes was abundant in the MSS metagenome. A previous study from our group [11] investigating As resistance genes in the MSS using a metagenomic approach also found that the *arrA* gene was the most diverse As gene in the sample, indicating that this dissimilatory arsenate reduction is the most frequent activity. This microbial reduction is one of the main pathways involved in As mobilization in anoxic environments because release of the most toxic and soluble form of As, AsIII, by reducing Fe- or Mn-oxides may increase the contamination of water bodies [66].

Microbial community physiological profile analysis based on the ability to use different carbon sources has been successfully used to characterize microbial diversity in different environments [67–69]. Xiong *et al*. [70] observed that soil uncontaminated by As showed greater metabolic diversity (C sources consumed) than soil newly contaminated with this metalloid, indicating that the microbial community was affected by this contamination. By contrast, our data showed a high metabolic diversity in the MSS, suggesting that As contamination is most likely not affecting the microbial diversity. Furthermore, other studies also reported that high nutrient concentrations in metal-contaminated sediments promote prokaryotic diversity [5, 71].

In freshwater ecosystems, phosphorus (P) and N are limiting nutrients, i.e., variation of these nutrient concentrations limits biological productivity. These nutrients were previously found in various organic and inorganic forms, and their bioavailability to higher trophic levels occurred through microbial transformations, because the organisms used them for growth and, in some cases, as an energy source [72].

The major transformations of N are N fixation, nitrification, denitrification, anammox, and ammonification, all highly dependent on the activities of a diverse assemblage of microorganisms such as bacteria, archaea, and fungi [73, 74]. In addition to metal contamination, Mina Stream is considered to be a eutrophic water body containing high concentrations of total N and its inorganic forms, nitrate (NO_3_^2^—N, 3103.8 μg l^-1^) and ammonium (NH_4_^+^-N, 829.5 μg l^-1^). Thus, it is likely that several bacterial and archaeal species related to the N cycle, such as *Thiobacillus denitrificans*, Candidatus *Nitrospira defluvii*, *Cenarchaeum symbiosum*, *Nitrosopumilus maritimus*, and Candidatus *Nitrosphaera gargensis*, among others, may play important roles in the N metabolism of the MSS. Candidatus *Nitrospira defluvii* was highly abundant in the MSS metagenome, being the bacterial genome with the highest coverage. These bacterial species are the dominant nitrite-oxidizing species in wastewater treatment plants and have already been found in metal-contaminated sediments [7, 75].

Analysis of N cycling genes from the MSS metagenome unveiled ammonium assimilation and ammonification as the two most abundant N cycle processes. Indeed, genes responsible for ammonium assimilation such as glutamate synthase (EC 1.4.1.13 and EC 1.4.1.14) and glutamine synthetase type I and type III (EC 6.3.1.2) were detected in our samples. Ammonium assimilation performed by the microbial community can retain N and make the sediment act as a temporary buffer in aquatic environments [76, 77]. The ammonification process is performed by saprophytic bacteria and is based on the decomposition of organic molecules containing N, e.g., amino acids and DNA that are released into the environment when an organism excretes waste or dies. N is required for the survival of all organisms, because it is an essential component of DNA, RNA, and protein, and thus, is essential for the maintenance of the aquatic microbial community. As most N exists in the form of organic molecules, the availability of N to higher trophic levels depends on microbial transformation.

In conclusion, our data reveal that the microbial communities from the MSS have significantly different features than those presented by other metal-contaminated environments. The data recovered agree with the expected assemblage of organisms thriving in metal-rich and eutrophic environments. This study provides important insights into the structure of the prokaryotic community of a tropical freshwater sediment, indicating a possible role for this community in the N and C cycles and in the transformation of Fe and As. Functional annotation unveiled a high degree of diversity of several metal resistance genes, indicating that this microbial community is well adapted to environments containing metal contamination. Finally, the results reported here expand the current knowledge of the microbial taxonomic and functional composition of tropical, metal-contaminated, freshwater sediments. Our data, together with those revealed by many other research efforts across the globe, may be an indirect and yet relevant contribution to the enormous endeavor being championed by the Earth microbiome project.

## Supporting Information

S1 Fig Rarefaction curve. Rarefaction curve of number of OTUs observed with an evolutionary distance of 0.03, 0.05 and 0.10.
(TIF)

S2 Fig Fragment recruitment plots of the MSS contigs by bacterial and archaeal genomes. The comparison was made using BLASTn. Vertical axis showed the % identity of the metagenomic contigs to the respective bacterial or archaeal genome. A—*Anaeromyxobacter dehalogenans* 2CP-1 (CP000251.1); B—*Chitinophaga pinensis* DSM2588 (CP001699.1); C—*Geobacter metallireducens* (CP000148.1); D—*Leptothrix chlolodnii* (CP001013.1); E—*Sideroxydans lithotrophicus* (CP001965.1); F—*Thiobacillus denitrificans* ATCC25259 (CP000116.1); G—*Thiomonas arsenitoxydans* 3As (FP475956.1); H—Candidatus *Nitrososphaera gargensis* (CP002408.1); I—*Cenarchaeum symbiosum* (DP000238.1).
(TIF)

S3 Fig The bacterial (A and B) and archaeal (C and D) standard curves (A). (TIF)

S4 Fig The C_t_ values from the 16S rRNA gene amplifications. A and B represent bacterial and archaeal amplifications, respectively.
(TIF)

S5 Fig Nitrogen cycle representation obtained in the Keeg Mapper analysis of MG RAST web server based on SEED database. The red square represents the presence of enzyme sequence in the MSS metagenome.
(TIF)

S1 Table Taxonomic affiliation of 16S rRNA gene OTUs based on Greengenes database. (XLSX)

S2 Table Carbon sources utilization by microbial communities in aerobic and anaerobic condition and diversity index in sediment of the Mina stream. (DOCX)
