# Supplementary figures and images for: Metagenome of a Microbial Community Inhabiting a Metal-Rich Tropical Stream Sediment
Source: PLoS One. 2015 Mar 5;10(3):e0119465. doi: 10.1371/journal.pone.0119465 (PMC4351183; doi:10.1371/journal.pone.0119465)

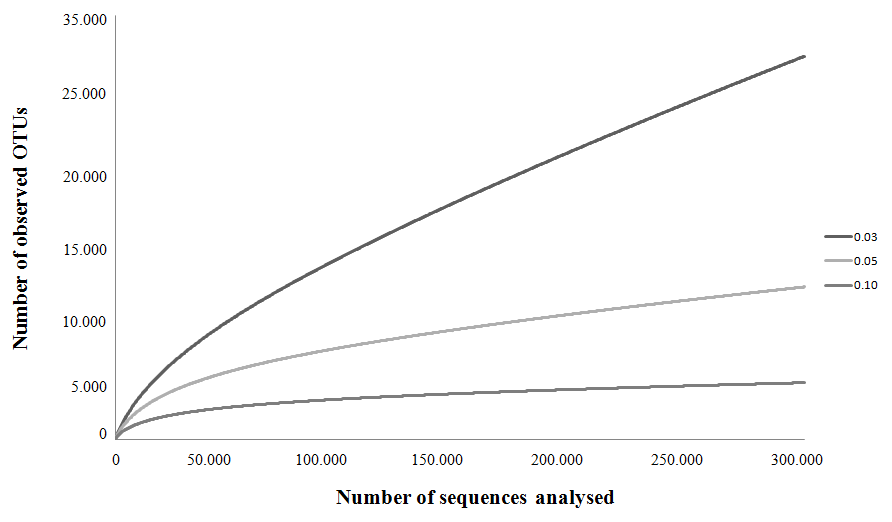

Supplement: S1 Fig — Rarefaction curve of number of OTUs observed with an evolutionary distance of 0.03, 0.05 and 0.10. (TIF) [file pone.0119465.s001.tif]

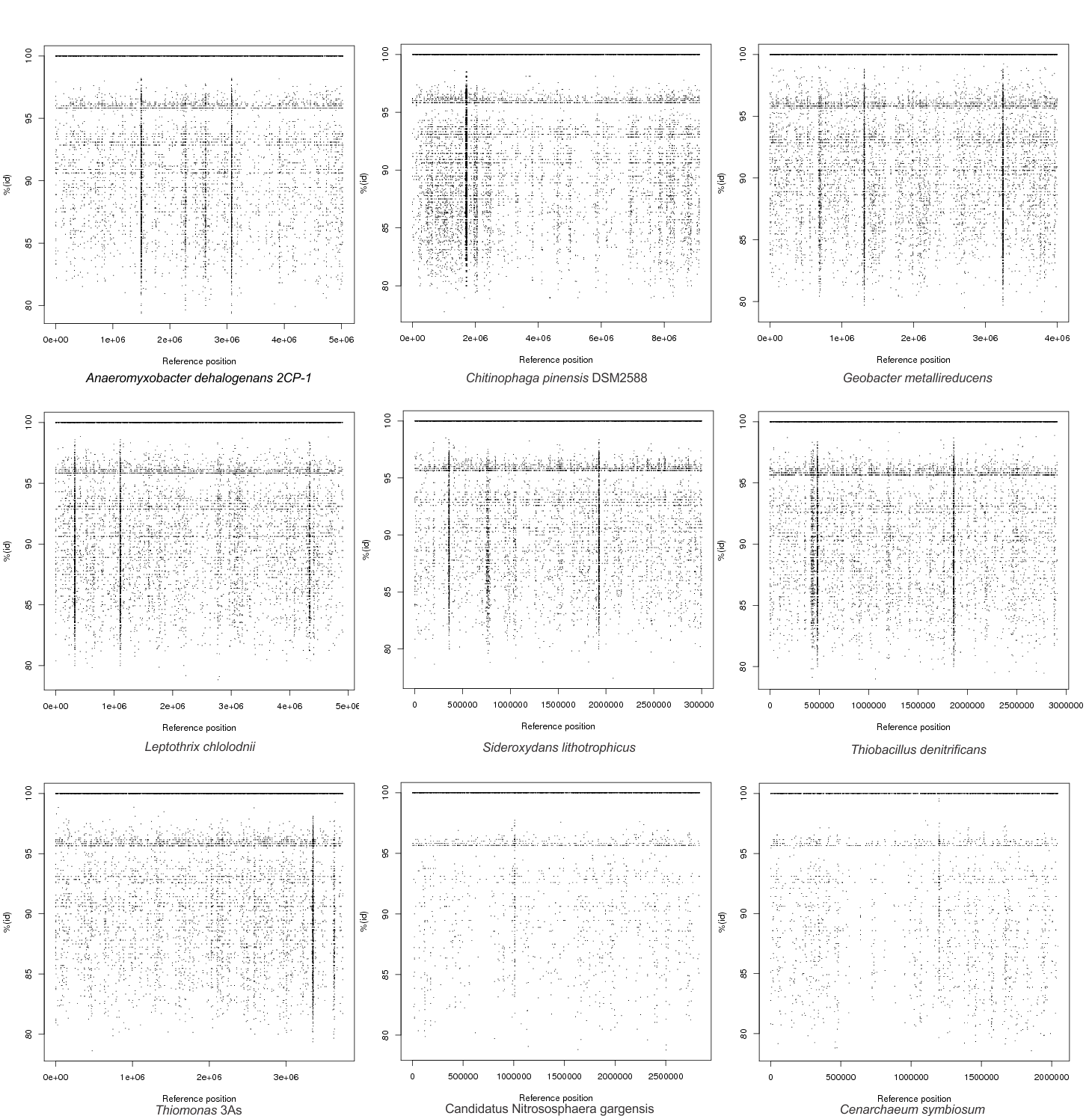

Supplement: S2 Fig — The comparison was made using BLASTn. Vertical axis showed the % identity of the metagenomic contigs to the respective bacterial or archaeal genome. A—Anaeromyxobacter dehalogenans 2CP-1 (CP000251.1); B—Chitinophaga pinensis DSM2588 (CP001699.1); C—Geobacter metallireducens (CP000148.1); D—Leptothrix chlolodnii (CP001013.1); E—Sideroxydans lithotrophicus (CP001965.1); F—Thiobacillus denitrificans ATCC25259 (CP000116.1); G—Thiomonas arsenitoxydans 3As (FP475956.1); H—Candidatus Nitrososphaera gargensis (CP002408.1); I—Cenarchaeum symbiosum (DP000238.1). (TIF) [file pone.0119465.s002.tif]

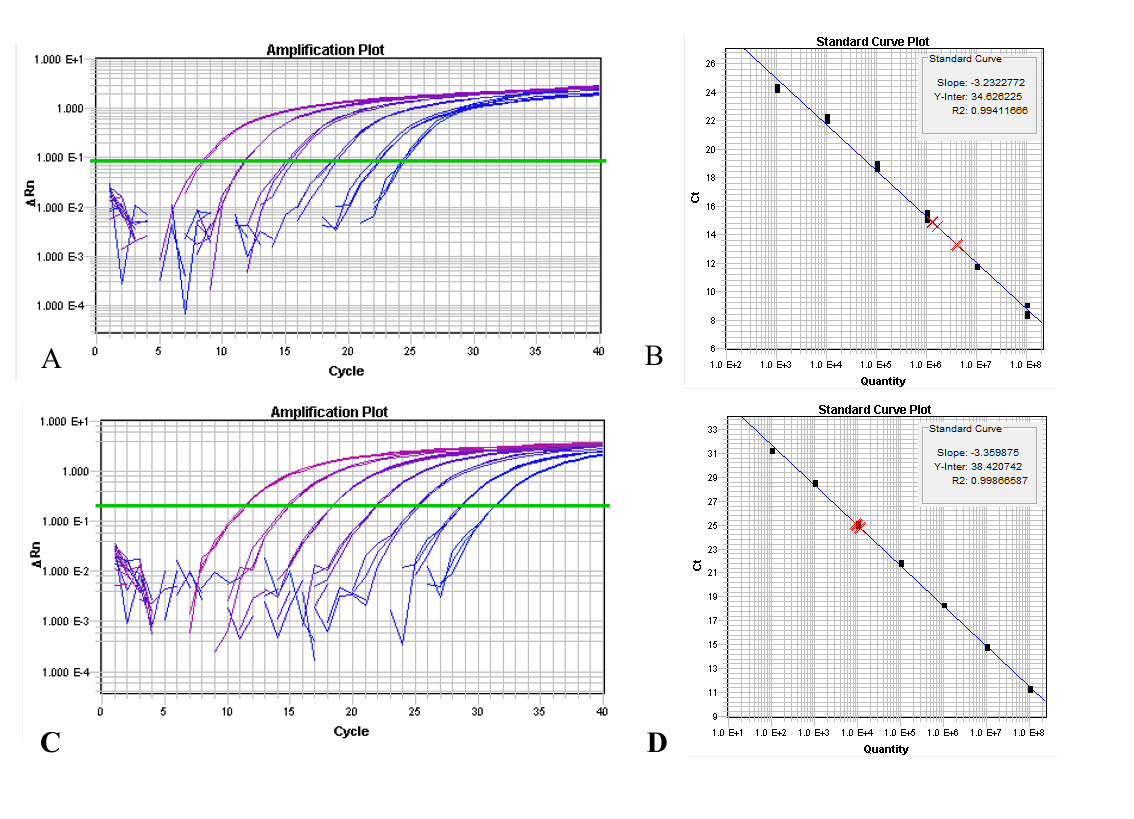

Supplement: S3 Fig — (TIF) [file pone.0119465.s003.tif]

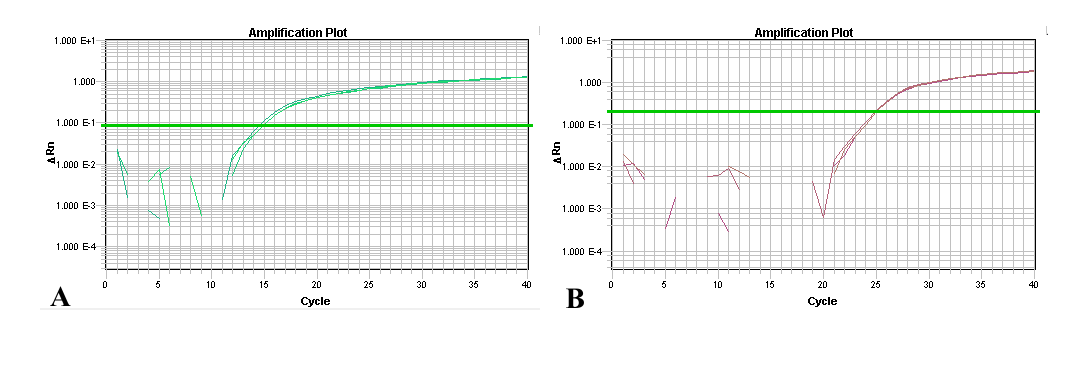

Supplement: S4 Fig — A and B represent bacterial and archaeal amplifications, respectively. (TIF) [file pone.0119465.s004.tif]

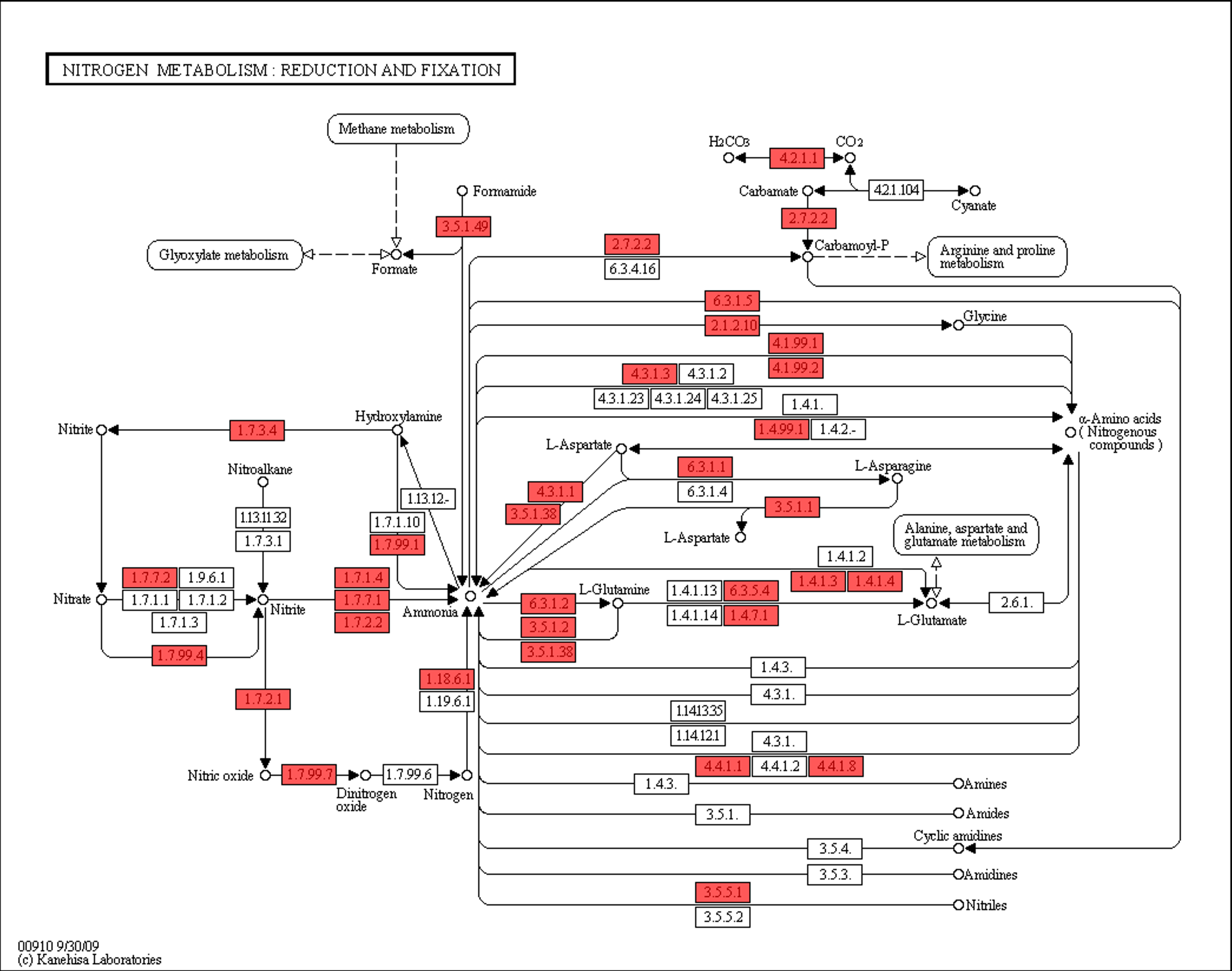

Supplement: S5 Fig — The red square represents the presence of enzyme sequence in the MSS metagenome. (TIF) [file pone.0119465.s005.tif]
