# Supplementary material for: Metagenome of a Microbial Community Inhabiting a Metal-Rich Tropical Stream Sediment
Source: PLoS One. 2015 Mar 5;10(3):e0119465. doi: 10.1371/journal.pone.0119465 (PMC4351183; doi:10.1371/journal.pone.0119465)
Supplement: S2 Table — (DOCX) [file pone.0119465.s007.docx]

S2 Table. Carbon sources utilization by microbial communities in aerobic and anaerobic condition and diversity index in sediment of the Mina stream.

| Carbon source | Grow condition | |
| --- | --- | --- |
|  | Aerobic | Anaerobic |
| β-Methyl-D-Glucoside | + | + |
| D-Galactonic Acid γ-Lactone | + | + |
| L-Arginine | + | + |
| Pyruvic Acid Methyl Ester | + | + |
| D-Xylose | + | + |
| D-Galacturonic Acid | + | + |
| L-Asparagine | + | + |
| Tween 40 | + | + |
| i-Erythritol | + | + |
| 2-Hydroxy Benzoic Acid | - | - |
| L-Phenylalanine | + | + |
| Tween 80 | + | + |
| D-Mannitol | + | + |
| 4-Hydroxy Benzoic Acid | + | + |
| L-Serine | + | + |
| α-Cyclodextrin | + | + |
| N-Acetyl-D-Glucosamine | + | + |
| γ- Hydroxybutyric Acid | + | + |
| L-Threonine | - | + |
| Glycogen | - | + |
| D-Glucosaminic Acid | + | + |
| Itaconic Acid | + | + |
| Glycyl-L-Glutamic Acid | + | + |
| D-Cellobiose | + | + |
| Glucose-1-Phosphate | + | + |
| α-Ketobutyric Acid | - | + |
| Phenylethylamine | + | + |
| α-D-Lactose | - | + |
| D,L-α-Glycerolphosphate | + | + |
| D-Malic acid | + | + |
| Putrescine | + | + |
| Richeness | 26 | 30 |
| Shannon- Weaver index | 2.96 | 3.37 |
| Simpson index | 0.9 | 0.99 |
